# Concurrent allergy and helminthiasis in underprivileged urban South African adults previously residing in rural areas

**DOI:** 10.1111/pim.12913

**Published:** 2022-03-02

**Authors:** Zilungile Lynette Mkhize‐Kwitshana, Pragalathan Naidoo, Ntombifikile M. Nkwanyana, Musawenkosi L. H. Mabaso

**Affiliations:** ^1^ 56394 Department of Medical Microbiology School of Laboratory Medicine and Medical Sciences College of Health Sciences Nelson R. Mandela Medical School Campus University of KwaZulu‐Natal Durban South Africa; ^2^ Research Capacity Development Division South African Medical Research Council Cape Town South Africa; ^3^ Discipline of Public Health Medicine School of Nursing and Public Health College of Health Sciences Howard College University of KwaZulu Natal Durban South Africa; ^4^ Human Sciences Research Council (HAST) Durban South Africa

**Keywords:** *Ascaris lumbricoides*, helminths and allergy, hygiene hypothesis, *Trichuris trichuria*

## Abstract

This study investigated whether prior exposure to helminths (*Ascaris* IgE, *Ascaris* eggs and *Trichuris* eggs) either in childhood or in adulthood, and residence in rural and resource‐limited urban areas influence allergy outcomes (asthma, rhinitis, IgE atopy and food allergy) in a South African population. Participants historical and present allergies data were collected through questionnaires and clinical record files. Coproscopy and immunoassays (ImmunoCAP^TM^ Phadiatop, total IgE and allergen‐specific fx3 IgE immunoassays and *Ascaris* IgE radioallergosorbent [RAST] tests) were used for active helminthiasis and allergy screens respectively. Data were analysed using logistic regression analysis, and models were adjusted for age, gender and locality. High *Ascaris* IgE was significantly associated with asthma (adjusted odds ratio [aOR] = 2.20, *p* = .047), IgE atopy (aOR = 18.18, *p* < .0001) and food allergy (aOR = 14.47, *p* < .0001). Asthma was significantly less likely among participants with *Ascaris* eggs (aOR = 0.43, *p* = .048) and *Trichuris* eggs (aOR = 0.36, *p* = .024). The findings of co‐occurrent helminthiasis and allergic disorders in a population that has resided both in rural and peri‐urban informal settlements both oppose and agree with two main notions of the hygiene hypothesis that (i) individuals residing in rural settings with poor sanitation and geohelminth infection are less prone to allergy, and (ii) helminth infections protect against allergy respectively. Further research is warranted.

## 1 INTRODUCTION

In the original ‘hygiene hypothesis’, Strachan proposed that increased microbial exposure in early life could protect children from developing immune hypersensitivities later in life.^1^ Decades later, the increase in allergic and autoimmune diseases in the industrialized world was also suggested to be associated with a decline in childhood infections due to effective vaccination, use of antibiotics, improved sanitation and personal hygiene.^2^ Immunologically, the concept was explained by the differential function of T helper (Th) cells with polarized cytokine profiles that counter‐regulate each other.^3^ This proposed a Th1‐dominant immune phenotype induced and imprinted by early life microbial exposures which was thought to inhibit the Th2‐atopic immunopathology.^4^ The simplistic Th1‐Th2 cross‐regulation theory was since challenged as it could no longer be explained in terms of why the Th2‐dominant response induced by helminth parasites were also proposed to protect against the Th2‐inflammatory allergic disorders, as well as why the Th1/Th17‐induced type 1 diabetes, Crohn's disease and multiple sclerosis were increasing in developed countries where, according to this theory, the decline in Th1 priming was supposedly associated with uncontrolled Th2‐associated increase in allergy.^5^, ^6^

Further refinement of the original hygiene hypothesis then brought two further theories suggesting the role of gut microbiome and intestinal parasites in shaping the development of the human immune system.^7^, ^8^, ^9^ These ‘microbial flora’ and ‘old friends’ theories suggest a role of early exposure to microbial and parasite infections, respectively, in the development of a balanced immune system that is able to respond appropriately to offensive antigens and not respond to innocuous ones.^8^, ^10^, ^11^ With regard to helminth parasites, extensive evidence of their protective effects against allergen reactivity in humans, as well as a series of mouse models of allergic diseases where helminths exhibited a protective role against a variety of allergic diseases, was put forward.^4^

A review by Briggs et al.^12^ highlighted the still‐unresolved role played by helminths in atopy and autoimmunity. On the one hand, extensive evidence suggesting that the infection with helminth parasites aggravates the inflammatory allergic and autoimmune diseases is juxtaposed to the evidence suggesting their protective role.^12^ Another theory proposes a species‐specific protection, wherein schistosomiasis is reported to be protective against atopy, and hookworm protects against asthma while ascariasis and trichuriasis are less protective.^2^ The latest modification of the hygiene hypothesis and helminths suggests that these organisms ameliorate the inflammatory (Th1/Th17‐autoimmune and Th2‐allergic) responses through increased regulatory T cells (Tregs) and their down‐modulatory cytokines IL‐10 and TGF‐β.^6^, ^13^ These highlight unresolved issues with regard to the explicit role of helminth parasites on protection versus exacerbation of immune disorders. Clinically, the use of live worms for the treatment of inflammatory bowel disease that had gained popularity previously is now also being challenged.^12^

It is also suggested that the life history of exposure is important, where early exposure is more beneficial compared with later in life.^14^ These leave a question as to whether present or prior helminth infections do have an impact on preventing the occurrence of immune disorders in affected hosts at any point in life, and if not, what are the pathological consequences of concurrent manifestations. Taking all this into consideration, the present study reports on the co‐occurrence of atopy, rhinitis, asthma, food allergy and intestinal helminths infections among adults, who had reported prior exposure to helminth parasites either in childhood or adulthood. The adults had previously resided in rural villages then relocated to a poorly resourced, city peri‐urban informal settlement.

## 2 METHODS

### 2.1 Study design

The study was approved by the South African Medical Research Council (SAMRC) and the University of Stellenbosch Ethics Committees (NO04/02/045). Permission to conduct the study was provided by the Matthew Goniwe Clinic management team. This cross‐sectional analysis used data obtained from the prospective study that were tracking the immune profile of adults during helminthic infection and anthelmintic treatment (n = 218), partly described in detail elsewhere.^15^, ^16^, ^17^

### 2.2 Study population and setting

Study participants were Xhosa adults (18 years and above) who were clinic attendees, presenting for different ailments, some were accompanying their relatives to the clinic and others were attending the clinic's HIV/AIDS support group. This is a Health District clinic of the Metro Region and is the largest in this region, servicing the informal settlement. These individuals were recruited by convenience sampling and participated in a larger prospective immunology and deworming study published in part elsewhere.^15^ With the exception of a few (approximately 10%, who were born in the City of Cape Town and elsewhere) the majority of the participants (approximately 90%) had moved from rural villages in the neighbouring Eastern Cape province of South Africa to a suburb of Cape Town within an informal settlement in the Western Cape province of South Africa. The area is poorly resourced, with lack of clean water and proper sanitation, overcrowded and previous reports showed high prevalence of helminth infections.^18^ Both *Ascaris lumbricoides* and *Trichuris trichuria* are the most predominant intestinal helminths in the Western Cape Province and Cape Town^19^ and Eastern Cape.^20^

### 2.3 Data collection

#### 2.3.1 Allergy and previous worm infection recall

Lifetime history and current experience of asthma and rhinitis symptoms and treatment as well as current use of asthma inhalers and rhinitis nasal sprays or other treatment were collected through questionnaires administered in the local isiXhosa language. Clinical records were also used to confirm clinician‐diagnosed allergies and treatment where available. Asthma and rhinitis recall and clinical diagnosis were also recorded based on the use of prescribed medications including inhalers such as Ventolin and its generics (e.g. asthavent, venteze etc.), nasal sprays such as Beclomethasone (Beconase) nasal spray and its generics and other rhinitis prescriptions also recorded from patient files to confirm asthma and rhinitis, respectively, where data were available in case record files, as well as those carrying these medications to the clinic at the time of interviewing.

Similarly, history of worm infection, as described in a previous study that enquired about history of intestinal helminth infection, more than 70% reported a recall of worm infection either as a child or an adult, more often both as a child or adult.^15^ Most had reported infection as a child before relocating from the rural home. The common practice of defecating in the open field, both in the rural villages and informal settlement made the experience of passing worms easy to recall and describe the worms. The description of the worms passed fitted mostly *A*. *lumbricoides* and a few others such as *T*. *trichuria* and tapeworm species. These data were elaborated in detail in a previous publication^15^ but for purposes of this work, this information is relevant as it qualitatively confirms the lifetime history (from childhood to adulthood) of worm exposure in this population, previously residing in rural areas then moved to poorly resourced informal settlements. This context also relates to the proposed concept early life or prior, and current helminth exposure and co‐occurrence of allergy in an under resourced population.

#### 2.3.2 Detection of current helminth infection

Both *A*. *lumbricoides* and *T*. *trichuria* are the most common helminths in the study area and were thus the focus of this study. Two stool samples, collected on two different days, were used for detection of helminth infection. Coproscopy, using Kato‐Katz and the modified formol‐ether concentration (Mini‐Parasep) by two independent microscopists, were used to detect the presence of helminth eggs. Mebendazole and praziquantel for schistosomiasis treatment were provided for all participants found to be infected with worms.

Serology was also used to increase the sensitivity of microscopy for intestinal helminth infection or exposure. *A*. *lumbricoides*‐specific IgE levels were thus analysed. Antibody cross reactivity with *T*. *trichuria* has been documented,^21^ and therefore this antibody is an appropriate proxy indicator of worm exposure for this setting where both these helminths are most common. However, the ability of ascariasis to increase allergic sensitization (atopy) is acknowledged, and in this section, the presence of high *Ascaris* IgE is used (in part, with stool parasitology) in the context of describing exposure/non‐exposure in order to classify those who had past or present helminthiasis infection. This analyte may be either a confounder or an intermediate factor in the association between helminth infection and allergy.

#### 2.3.3 Biochemical measures and allergy screening

The primary exposure variables were faecal *A*. *lumbricoides* and/or *T*. *trichiuria* egg excretion plus serum *A*. *lumbricoides* ‐specific IgE (used as a proxy to indicate helminth exposure) as well as demographic variables such as age, gender and locality type (place of birth). The study biological outcomes included eosinophil counts, serum total IgE, total IgE, defined as overall quantity of all immunoglobulin E subtypes in blood. High total IgE for the study methodology are levels equal to or above the defined cut‐off value of 70 kU/L. Allergic disorder outcome variables included four measures: (1) IgE atopy (a positive test for evidence of allergen sensitization by specific IgE using Phadiatop), (2) asthma, (3) rhinitis and (4) food allergy (Table 1). Allergic sensitization or atopy was screened using the ImmunoCAP^TM^ Phadiatop assay. Positive allergen IgE reactivity was measured on discs containing locally relevant inhalant allergens (dog and cat epithelium, house dust and house dust mites and cockroaches); mixed trees (olive, willow, pine, eucalyptus, acacia and melaleuca); mixed weeds (marguerits, dandelion, plantain, goosefoot and golden rod); Bermuda and Timothy grass and mixed mould (*Penicillium notatum*, *Cladosporium herbarum*, *Aspergillus fumigatus* and *Alternaria aternata*). Total serum IgE, food sensitivity and *A*. *lumbricoides*‐specific IgE levels were determined by ImmunoCAP Total IgE, ImmunoCAP allergin‐specific fx3 IgE, and Ascaris IgE radioallergosorbent (RAST) tests respectively. Food allergens included egg whites, milk, soya, peanuts, wheat and fish (cod and shrimp).

**TABLE 1:** Primary exposure and proxy variables for parasitic infections and allergic outcomes used in the analysis

Variables	Description of measures	Reference ranges and variable coding
Helminth exposure		
*Ascaris* IgE	Antibody isotype immune responses to *A*. *lumbricoides*	(Present) or high IgE ≥0.35 kU/L = 1 (Absent) or low/normal IgE <0.35 kU/L = 2
*Ascaris* eggs	Eggs detected in stool specimens	Yes = 1 No = 2
*Trichuris* eggs	Eggs detected in stool specimens	Yes = 1 No = 2
Allergy‐related disorders	Asthma and rhinitis	Yes = 1 No = 2
Total IgE	Serum total IgE	Low <70 kU/L = 2 High ≥70 kU/L = 1
Eosinophils	Pro‐inflammatory white blood cells for parasitic infections or allergic reactions	Low/normal <0.35 × 10^6^ cells/ml = 2 High ≥0.35 × 10^6^ cells/ml = 1
IgE Atopy	Allergen‐antigen‐specific immunoglobulin E measurements	Allergic ≥0.175 kU/L = 1 Non‐allergic <0.175 kU/L = 2
IgE fx3	Food‐antigen‐specific immunoglobulin E measurements	Allergic ≥0.175 kU/L = 1 Non‐allergic <0.175 kU/L = 2
Asthma recall	Participant's description of ever experiencing symptoms indicative of asthma such cough, wheeze, shortness of breath and chest tightness and the isiXhosa name ‘umbefu’ and/or current/past clinician prescription of medication for such, at any stage in life	Yes = 1 No = 2
Rhinitis recall	Participant description of ever experiencing symptoms indicative of rhinitis such as sneezing, itching, nasal congestion, runny nose and postnasal drip; and the isiXhosa name for rhinitis ‘umfinxizo’ and/or current/past clinician prescription of medication for such at any stage in life	Yes = 1 No = 2

Abbreviations: IgE fx3, immunoglobulin E food allergy screen; IgE, immunoglobulin E.

#### 2.3.4 Measures

Primary outcome variables of allergy‐related disorders included four measures: asthma, rhinitis, IgE atopy and food allergy (IgE Fx3). Exposure variables included helminth parasitic infection exposure variables: serum *A. lumbricoides*‐specific IgE, stool *A. lumbricoides* and *T. trichuria* eggs excretion; as well as demographic variables such as age, gender and locality type (place of birth) (Table 1).

### 2.4 Statistical analysis

Descriptive statistics frequencies and percentages were used to summarize the data. Pearson chi‐square test was used to test for differences between categorical variables. Bivariate and multivariate logistic regressions were used to analyse the association between measures of allergy‐related disorders and helminth parasitic infection exposure. Crude (OR) and adjusted odds ratios (aOR)) and 95% confidence intervals (95% CI) with a *p*‐value ≤.05 were used to determine the level of statistical significance. Data were analysed using STATA version 15.0 (Stata Corporation).

## 3 RESULTS

### 3.1 Characteristics of the study population

Table 2 highlights the demographics and biological characteristics of the study population. A third of participants were aged 30–39 years (34.4%), and the majority were females (86.2%). Total IgE levels were generally high, with a mean serum total IgE of 419.5 kU/L (reference value is <70 kU/L) with a high proportion of study participants having a high total IgE (64.2%), and 19.3% had high eosinophil counts. Regarding helminthiasis, a quarter had active worm infection as shown by *Ascaris* (24.1%) and *Trichuris* (24.8%) eggs in stool and 33.9% had high *Ascaris* IgE. The overall prevalence of helminth infection was 43%, by egg excretion positivity; however, the prevalence as measured by either high *Ascaris*‐specific IgE, and/or *Trichuris*/*Ascaris* egg excretion was 66% in this sample population. In terms of allergic disorders, overall, asthma, rhinitis, IgE atopy and food allergy prevalence were 17.5%, 54.7%, 39.9% and 11.5% respectively (Table 2).

**TABLE 2:** Characteristics of study participants including proxy variables for parasitic infections (n = 218)

Variables	N	%
Age groups in years		
18–25	37	17.5
25–29	56	26.4
30–39	74	34.9
40+	45	21.2
Gender		
Male	30	13.8
Female	188	86.2
Province		
Cape Town	18	8.3
Eastern cape	193	88.5
Others	7	3.2
Total IgE		
Low (<70 kU/L)	78	35.8
High (>70 kU/L)	140	64.2
Eosinophils		
Low (<0.35 cells/ml)	176	80.7
High (>0.35 cells/ml)	42	19.3
*Ascaris* IgE		
Low (<0.35 kU/L)	144	66.1
High (≥0.35 kU/L)	74	33.9
*Trichuris* eggs		
Yes	48	24.8
No	152	75.2
*Ascaris* eggs		
Yes	49	24.1
No	154	75.9
Asthma		
Yes	37	17.5
No	175	82.5
Rhinitis		
Yes	116	54.7
No	96	45.3
IgE atopy		
Yes	87	39.9
No	131	60.1
Food allergy		
Yes	25	11.5
No	193	88.5

Note

Subtotals are not always equal due to some missing data.

Abbreviation: IgE, immunoglobulin E.

The majority of participants (88.5%) had migrated from rural villages in the Eastern Cape (their place of birth) to the Western Cape Province's City of Cape Town informal settlement. Tables 3 and 4 show the prevalence of helminth exposure and allergy‐related disorders, respectively, in relation to participants’ place of birth (Cape Town, Eastern Cape and other Provinces). A significant association between participants’ place of birth and (i) helminth exposure (high *Ascaris* IgE: χ^2^ *p* = .002 and *Trichuris* eggs: χ^2^ *p* < .0001) and (ii) allergy‐related disorder outcomes (IgE atopy: χ^2^ *p* = .006 and food allergy: χ^2^ *p* = .007) were observed. Those born in Cape Town had the highest prevalence of high *Ascaris* IgE (66.7%) and *Trichuris* eggs (83.3%) while *Ascaris* eggs were most prevalent in participants originally from the Eastern Cape (26%). Asthma prevalence was similar across all the places of birth and ranged between 16% and 18%. The Eastern Cape had the highest prevalence of rhinitis (55.6%) while IgE atopy (72.2%) and food allergy (33.3%) was most prevalent among those born in Cape Town (Tables 3 and 4; Figures S1 and S2).

**TABLE 3:** Prevalence of helminth exposure stratified according to place of birth (Cape Town, Eastern Cape and other Provinces) (n = 218)

Variables		High *Ascaris* IgE (n = 74)		*Ascaris* eggs (n = 49)		*Trichuris* eggs (n = 48)	
		Yes (≥0.35 kU/L)	No (<0.35 kU/L)	Yes	No	Yes	No
Cape Town (n = 18)	n (%)	12 (66.7)	6 (33.3)	3(16.7)	15 (83.3)	15 (83.3)	3 (16.7)
Eastern Cape (n = 193)^a^ (n = 177)^b^	n (%)	62 (32.1)	131 (67.9)	46 (26)	131 (74)	30 (16.9)	147 (83.1)
Other Provinces (n = 7)	n (%)	0 (0)	7 (100)	0 (0)	7 (100)	3 (42.8)	2 (28.6)
	χ^2^ *p*‐value	.002		.213		<.0001	

Note

Subtotals are not always equal due to some missing data. χ^2^: chi‐square test *p*‐value.

Abbreviation: IgE, immunoglobulin E.

^a^ Denotes sample size for high Ascaris IgE.

^b^ Denotes sample size for Ascaris eggs and Trichuris eggs.

**TABLE 4:** Prevalence of allergy‐related disorders stratified according to place of birth (Cape Town, Eastern Cape and other Provinces) (n = 218)

Variables		Asthma (N = 212)		Rhinitis (N = 212)		IgE atopy (N = 218)		Food allergy (N = 218)	
		Yes	No	Yes	No	Yes	No	Yes	No
Cape Town (n = 17)^a^ (n = 18)^b^	n (%)	3 (17.6)	14 (82.4)	8 (47.1)	9 (52.9)	13 (72.2)	5 (27.8)	6 (33.3)	12 (66.7)
Eastern Cape (n = 189)^a^ (n = 193)^b^	n (%)	33 (17.5)	156 (82.5)	105 (55.6)	84 (44.4)	73 (37.8)	120 (62.2)	19 (9.8)	174 (90.2)
Other Provinces (n = 6)^a^ (n = 7)^b^	n (%)	1 (16.7)	5 (83.3)	3 (50)	3 (50)	1 (14.3)	6 (85.7)	0 (0)	7 (100)
	(χ^2^) *p*‐value	.999		.775		.006		.007	

Note

Subtotals are not always equal due to some missing data. χ^2^: chi‐square test *p*‐value.

Abbreviation: IgE, immunoglobulin E.

^a^ Denotes sample size for asthma and rhinitis.

^b^ Denotes sample size for IgE Atopy and food allergy.

### 3.2 Concurrent occurrence of helminth exposure and allergy‐related outcomes

**FIGURE 1:**
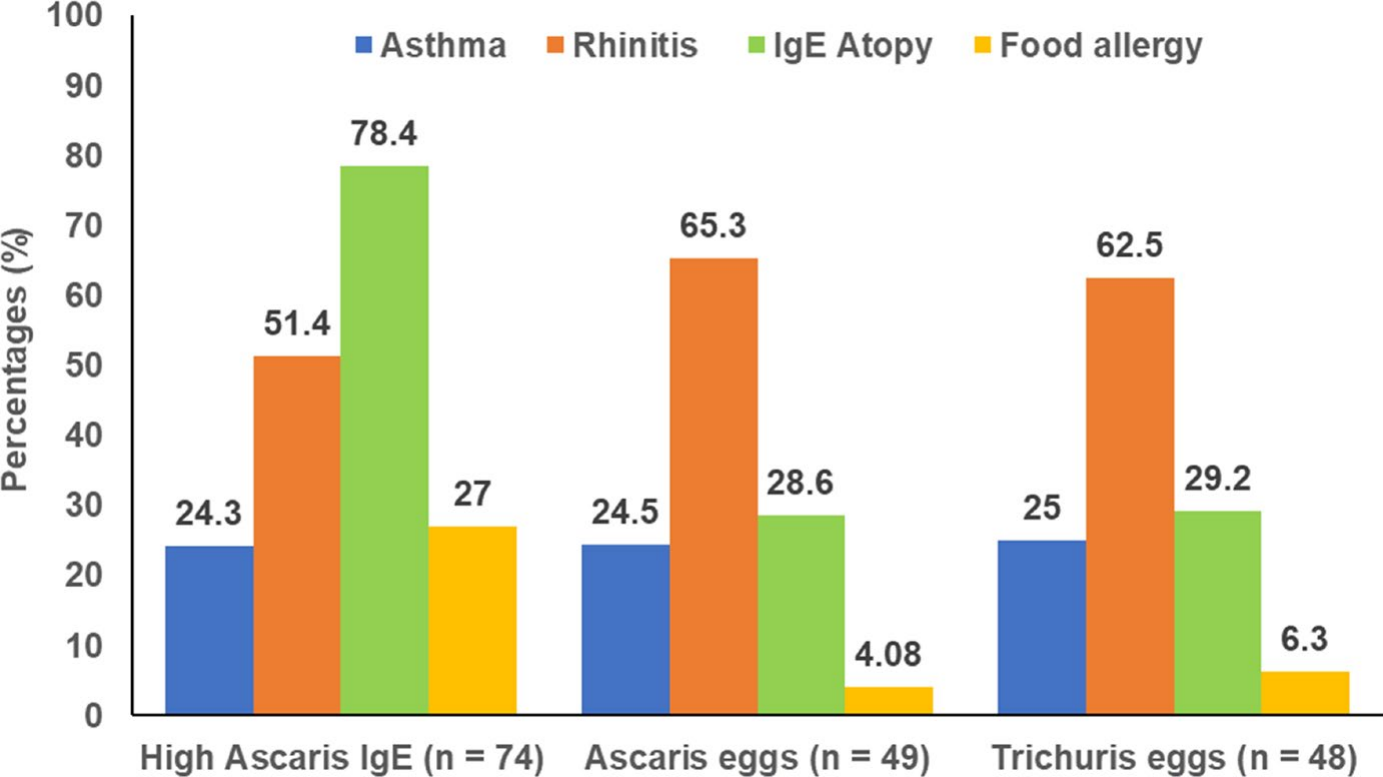
Prevalence of allergy outcomes in the different helminth exposure groups

Overall, IgE atopy (78.4%), rhinitis (51.4%) and food allergy (27%) were most prevalent among the group with high *Ascaris* IgE. Rhinitis prevalence was similar and higher in participants that had active worm infection as shown by stool positivity for *Ascaris* eggs (65.3%) and *Trichuris* eggs (62.5%). All helminth exposed groups (high *Ascaris* IgE and positive stool *Ascaris* and *Trichuris* eggs) had a similar prevalence of asthma ranging between 24.3%, 24.5 and 25% respectively (Figure 1).

### 3.3 Association between participants’ demographic characteristics and helminth exposure with allergy‐related outcomes

Males had a higher prevalence of asthma (*p* < .001), IgE atopy (*p* < .001) and food allergy (*p* < .001). The biological reactants, eosinophilia and high total serum IgE are characteristic biological markers associated with both exposure variables (parasitic infections) and outcome variables (allergy‐related disorders). As expected, participants with high total IgE levels significantly associated with allergy‐related disorders (IgE atopy: *p* < .001 and food allergy: *p* < .001). Likewise, high eosinophil levels were associated with asthma: *p* < .001, rhinitis: *p* = .012, IgE atopy: *p* = .001 and food allergy: *p* = .024 (Table 5).

**TABLE 5:** Allergy‐related disorders by demographic and biological characteristics

Variables	Asthma			Rhinitis			IgE Atopy			Food allergy		
	N	n (%)	*p*‐value	N	n (%)	*p*‐value	N	n (%)	*p*‐value	N	n (%)	*p*‐value
Age group in years												
18–25	36	5 (13.9)	.114	36	14 (38.9)	.096	37	17 (45.9)	.691	37	4 (10.8)	.370
25–29	54	5 (9.3)		54	33 (61.1)		56	21 (37.5)		56	6 (10.7)	
30–39	73	12 (16.4)		73	45 (61.6)		74	26 (35.1)		74	11 (14.9)	
40+	44	12 (27.3)		44	22 (50.0)		45	19 (42.2)		45	2 (4.4)	
Gender												
Male	29	12 (41.4)	<.001	29	19 (65.5)	.209	30	24 (80.0)	<.001	30	10 (33.3)	<.001
Female	183	25 (13.7)		183	97 (53.0)		188	63 (33.5)		188	15 (8.0)	
Total IgE												
Low (<70 kU/L)	75	10 (13.3)	.242	75	41 (54.7)	.991	78	9 (11.5)	<.001	78	0 (0)	<.001
High (>70 kU/L)	137	27 (19.7)		137	75 (54.7)		140	78 (55.7)		140	25 (17.9)	
Eosinophils												
Low (<0.35 cells/ml)	172	21 (12.2)	<.001	172	87 (50.6)	.012	176	61 (34.7)	.001	176	16 (9.1)	.024
High (>0.35 cells/ml)	40	16 (40.0)		40	29 (72.5)		42	26 (61.9)		42	9 (21.4)	

Note

Subtotals are not always equal due to some missing data.

Abbreviation: IgE, immunoglobulin E.

Table 6 shows the association between helminth exposure and allergy‐related outcomes. Models adjusted for age, gender and locality show that asthma was significantly less likely among those with *Ascaris* eggs (aOR = 0.43, 95% CI: 0.24–0.99, *p* = .048) and those with *Trichuris* eggs (aOR = 0.36, 95% CI: 0.15–0.87, *p* = .024) but more likely among those with high *Ascaris* IgE (aOR = 2.20, 95% CI: 0.66–3.75, *p* = .047). IgE atopy was significantly more likely among those with high Ascaris IgE (aOR = 18.18, 95% CI: 8.02–41.19, *p* < .0001). Food allergy was also significantly more likely among those with high *Ascaris* IgE (aOR = 14.47, 95% CI: 4.17–50.14, *p* < .0001).

**TABLE 6:** Allergy‐related disorders by helminth exposure

Helminth exposure		Allergy‐related disorders							
		Asthma		Rhinitis		IgE atopy		Food allergy	
		Yes	No	Yes	No	Yes	No	Yes	No
High *Ascaris* IgE	High (≥0.35 kU/L), n (%)	18 (24.3)	56 (75.7)	37 (51.4)	35 (48.6)	58 (78.4)	16 (21.6)	20 (27.0)	54 (73.0)
	Not high (<0.35 kU/L) (Reference group), n (%)	18 (14.6)	105 (85.4)	79 (56.4)	61 (43.6)	28 (19.6)	115 (80.4)	4 (2.8)	139 (97.2)
*p*‐value		.1268		.560		<.0001		<.0001	
OR (95% CI)		1.88 (0.90–3.89)		0.82 (0.46–1.44)		14.89 (7.46–29.71)		12.87 (4.20–39.40)	
*p*‐value		.047		.446		<.0001		<.0001	
OR (95% CI)		2.20 (0.66–3.75)		0.77 (0.40–1.49)		18.18 (8.02–41.19)		14.47 (4.17–50.14)	
*Ascaris* eggs	Yes, n (%)	12 (24.5)	37 (75.5)	32 (65.3)	17 (34.7)	14 (28.6)	35 (71.4)	2 (4.1)	47 (95.9)
	No (Reference group), n (%)	23 (15.5)	125 (84.5)	78 (52.7)	70 (47.3)	63 (41.4)	89 (58.6)	20 (13.2)	132 (86.8)
*p*‐value		.195		.138		.129		.310	
OR (95% CI)		1.76 (0.80–3.88)		1.69 (0.86–3.31)		0.57 (0.28–1.14)		0.28 (0.06–1.25)	
*p*‐value		.048		.054		.323		.530	
OR (95% CI)		0.43 (0.24–0.99)		0.49 (0.24–1.01)		0.49 (0.24–1.01)		0.64 (0.16–2.54)	
*Trichuris* eggs	Yes, n (%)	12 (25.0)	36 (75.0)	30 (625)	18 (37.5)	14 (29.2)	34 (70.8)	3 (6.3)	45 (93.8)
	No (Reference group), n (%)	23 (15.4)	126 (84.6)	80 (53.7)	69 (46.3)	63 (41.2)	90 (58.8)	19 (12.4)	134 (87.6)
*p*‐value		.135		.319		.173		.297	
OR (95% CI)		1.83 (0.83–4.03)		1.44 (0.74–2.80)		0.59 (0.29–1.19)		0.47 (0.13–1.66)	
*p*‐value		.024		.500		.563		.666	
OR (95% CI)		0.36 (0.15–0.87)		0.79 (0.39–1.58)		0.80 (0.37–1.71)		0.74 (0.19–2.92)	

Note

Subtotals are not always equal due to some missing data.

Abbreviations: aOR, adjusted odds ratio (adjusted for age, gender and locality); IgE, immunoglobulin E.

## 4 DISCUSSION

This study investigated the effects of exposure to *A*. *lumbricoides* (and by proxy, *T. trichuria*) and egg excretion on allergic disorders including atopy, asthma, food allergy and rhinitis in participants from a socioeconomically deprived rural population that had moved to urban informal areas.^15^ In the present study, the overall prevalence of ascariasis and trichuriasis was 43% as measured by egg excretion only and 66% by egg excretion plus high *Ascaris*‐specific IgE. More than 20% had active worm infection shown by *Ascaris* (24.1%) and *Trichuris* (24.6%) eggs in stool and approximately a third (33.9%) had high *Ascaris*‐specific IgE. Overall occurrence of allergic disorders in the study sample ranged between 11.5% (food allergy) and 39.9% (atopy), as measured by allergen specific IgE screen; and asthma (18%) and 55% rhinitis according to recall of disease symptoms and treatment. The study population generally had high serum total IgE. This is expected since helminth infections induce a polyclonal stimulation of IgE synthesis via IL‐4,^22^ giving rise to high serum total IgE. During assay of the latter and *Ascaris*‐specific IgE, there could be cross‐reactivity.

Regarding the concurrent manifestation of allergic disorders and helminth exposure, the majority (78.4%) of those with high *Ascaris* IgE also had high IgE atopy, and more than 50% had rhinitis. Those with active worm infection had more than 60% rhinitis, and all helminth exposed groups had more than 20% prevalence of asthma. In the present study, high *Ascaris* IgE showed a significant association with atopy and food allergy, both of which were increased after adjustment for demographic characteristics. Likewise, high *Ascaris* IgE was also significantly associated with asthma but significantly less likely among those with *Ascaris* and *Trichuris* eggs in stool (active worm infection). These results present high prevalence of both helminth infection and allergic disorders, and concurrent manifestation of both, in a population that has moved from rural villages into an urban informal settlement, with a history of childhood and/or adult worm infection and current evidence of worm infection. These findings do not support the hypothesis of an inverse relationship between parasite infection and allergic sensitization.^23^, ^24^, ^25^, ^26^

Contrary to previous studies that have suggested that helminth parasites protect against atopy,^27^, ^28^, ^29^, ^30^ in our study, multivariate analysis showed that individuals with high *Ascaris*‐specific IgE were fourteen times more likely to be atopic. This association was highly statistically significant. The significant association between atopy and high *Ascaris* IgE is a typical reflection of the documented presence of elevated *Ascaris* IgE being a risk factor for atopy or a genetic tendency towards allergic sensitization and asthma or wheezing.^29^

Other findings showed that male gender was associated with *Ascaris* infection. In addition, active worm infection (*Ascaris* and *Trichuris* eggs in stool) was also significantly associated with males, although they only constituted a smaller proportion (14%) of the study sample. This is in keeping with the general finding that males are generally more susceptible to parasite infections.^31^, ^32^

The findings of the current study also add another layer of ambiguity to the rephrased hygiene hypothesis that incorporates the acquisition of ‘old friends’ such as helminths, and microbiota early in life as primers of immunoregulation that results in an appropriately responsive immune response later in life.^8^ The individuals in the study had recalled childhood worm infection, while also growing up in rural settings, as the majority were born in rural Eastern Cape villages. Seventy percent of these adult participants had reported previous exposure to helminths in childhood and/or as adults, the majority of whom had moved to the city but within resource‐constrained informal settlement less than 10 years before, after previous residence in a rural setting.^15^ However, a significant proportion of these individuals also had allergic disorders.

These findings both oppose and agree with two main notions of the hygiene hypothesis that (i) individuals residing in rural settings with poor sanitation and geohelminth infection are less prone to allergy^33^ and (ii) helminth infections protect against allergy.^5^, ^6^ However, this study shows that those with high *Ascaris* IgE were significantly more likely to have asthma, food allergy and atopy, opposing the first point. Nonetheless, those with active worm infection were found to be significantly less likely to have asthma, which is in agreement with the second point. Some epidemiological reports suggest that allergy risk is reduced in high geohelminth infection settings as is the case with the current study area.^29^, ^34^

There are contradictory reports on the occurrence of allergic diseases in low‐ and middle‐income countries; however, urban areas are reported to be most affected than rural areas.^35^ Urbanization has been shown to cause allergy through air pollution and higher exposure to allergens and endotoxin.^30^, ^36^ Individuals in the current study have lived in rural settings as well as urban informal settlements with limited sanitation and clean water. The notions that reduced infections due to improved health care and personal hygiene and decreased exposure to helminths in the urban environment suggested to lead to insufficient stimulation of the immune system and increased susceptibility to allergy^37^ are discordant with our findings.

However, the study has several limitations. Firstly, it is limited by an interviewer‐administered, retrospective questionnaire which may have possible recall bias for asthma and rhinitis. However, there were several steps in mitigating some of these limitations. For example, in addition to the fact that for the history of worm infection, the worms described mostly resembled *Ascaris*, due to their conspicuous size, egg excretion and *Ascaris*‐specific IgE were also measured and confirmed high prevalence of the two helminth species. Likewise, for the history of asthma and rhinitis symptoms and treatment, current symptomatology and treatment were also recorded and confirmed through clinic records to mitigate the recall and self‐reporting bias. In addition, for allergic disorders, blood measurements for IgE atopy and food allergy as well as total and *Ascaris*‐specific IgE were undertaken. The study was also limited by the small sample size which is reflected by the wide confidence intervals in some of the associations. An additional confounder was gender bias since the majority of participants were females. This was a default representative of a healthcare sample, as is a common phenomenon where females are more likely to be healthcare seekers than males. Demographically, most participants were born in the Eastern Cape villages.

A spurious finding was the significant negative association between *Ascaris* egg excretion and asthma. During the early phase of *Ascaris* infection, the larvae migrate to the lungs before being coughed up, causing an eosinophilic pneumonia‐like symptom that may present as asthma^38^ A possible explanation could be that asthma is a heterogenous disease which includes atopic and non‐atopic forms.^39^ In particular, the non‐atopic form is more prevalent among older people, as similar to the age of the population studied here. The present study, however, did not distinguish or control for these different endotypes since the objective was to record any form of asthma in the presence or absence of helminthiasis. Others suggest that chronic but not acute helminth infections protect against allergy.^29^ In our study, active worm infections that were negatively associated with asthma cannot be classified as either chronic or acute. Further study with adequate sampling and power is required.

Although limited by several factors, the study highlights that early and current infection with helminths and exposure to rural and later, unhygienic environments (in the city informal settlements) do not preclude the occurrence of allergies induced by food and inhalant allergens, and asthma and rhinitis.

## 5 CONCLUSION

We have shown more than 20% concurrent active worm infection and allergy in adults with evidence of lifelong exposure to environmental stimulation factors (rural and informal settlements with lack of clean water and proper sanitation) as well as helminthiasis. This finding seems to be in agreement with the proposition that complex factors interact to determine the occurrence of allergic and autoimmune diseases in the context of helminth infections. This report adds to the ever‐contradictory studies of the interactions between helminths and hyperimmune disorders. More insights into the extension of the reformulated hygiene hypothesis and old friends’ theory are needed^40^ which require more in‐depth studies in different geographic settings. This knowledge is important for better diagnosis, treatment and prevention of allergy‐related diseases and the potential therapeutic or exacerbating significance of helminthiasis. Therefore, more studies are needed in order to gain a better understanding of this phenomenon.

## CONFLICT OF INTEREST

The authors declare that they have no known competing financial interests or personal relationships that could have appeared to influence the work reported in this paper.

## AUTHOR CONTRIBUTIONS

Zilungile Lynette Mkhize‐Kwitshana contributed to conceptualization, laboratory and field work and writing—original draft preparation. Pragalathan Naidoo contributed to conceptualization and writing—reviewing and editing. Ntombifikile M. Nkwanyana contributed to data analysis and validation. Musawenkosi L. H. Mabaso contributed to conceptualization, data analysis and writing—reviewing and editing.

## Supporting information

Fig S1‐S2

## Data Availability

The data that support the findings of this study are available from the corresponding author upon reasonable request.
