# Supplementary material for: Concurrent allergy and helminthiasis in underprivileged urban South African adults previously residing in rural areas
Source: Parasite Immunol. 2022 Mar 2;44(4-5):e12913. doi: 10.1111/pim.12913 (PMC9539504; doi:10.1111/pim.12913)
Supplement: Supplementary file 1 — Fig S1‐S2 [file PIM-44-e12913-s001.docx]

**Supplementary Figures S1 and S2**

**Supplementary Figure S1** and **Figure S2** show the prevalence of helminth exposure and allergy-related disorders in relation to participants place of birth.


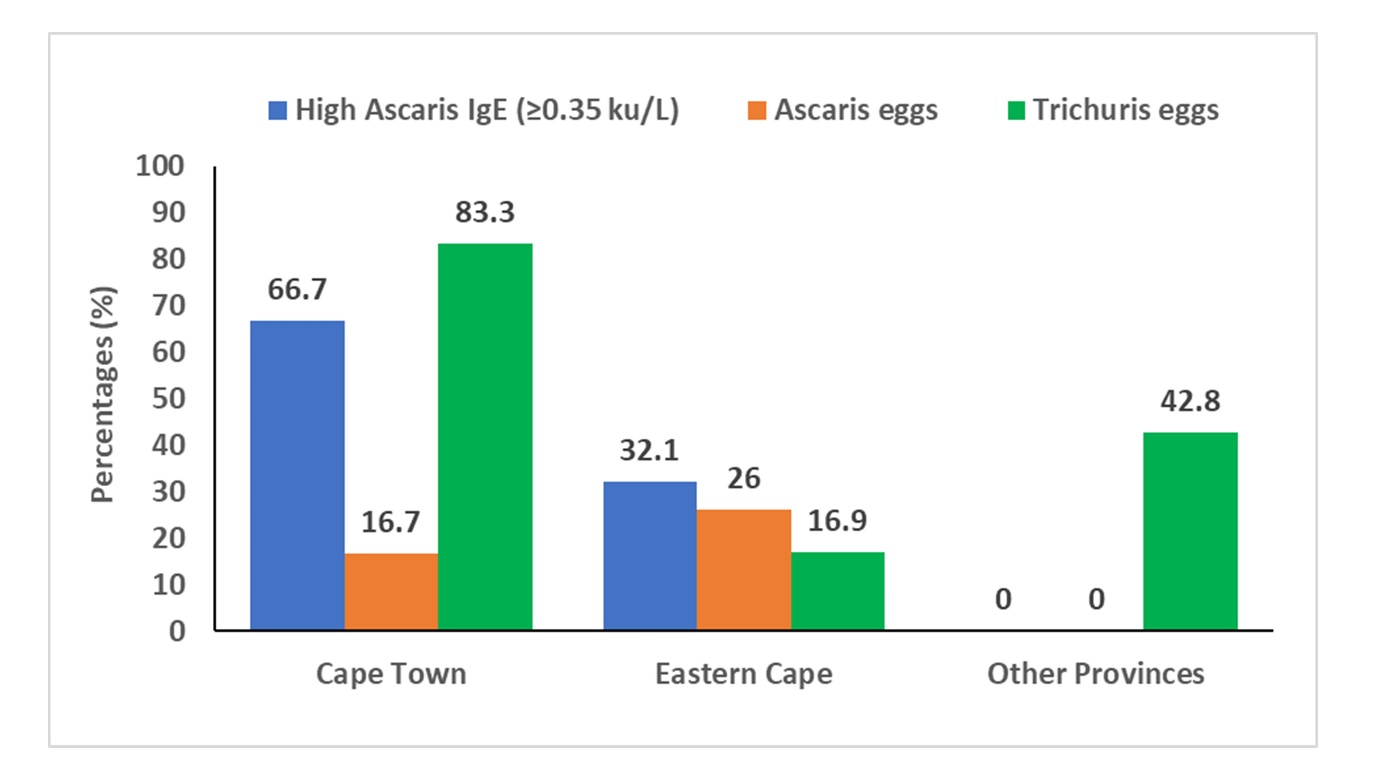
**Supplementary Figure S1. Prevalence of helminth exposure categorized according to participants place of birth.**

**Figure S1 footnote: Cape Town (n = 18). Eastern Cape (n = 193 for high Ascaris IgE; n = 177 for Ascaris eggs and Trichuris eggs). Other Provinces (n = 7). IgE: immunoglobulin E.**


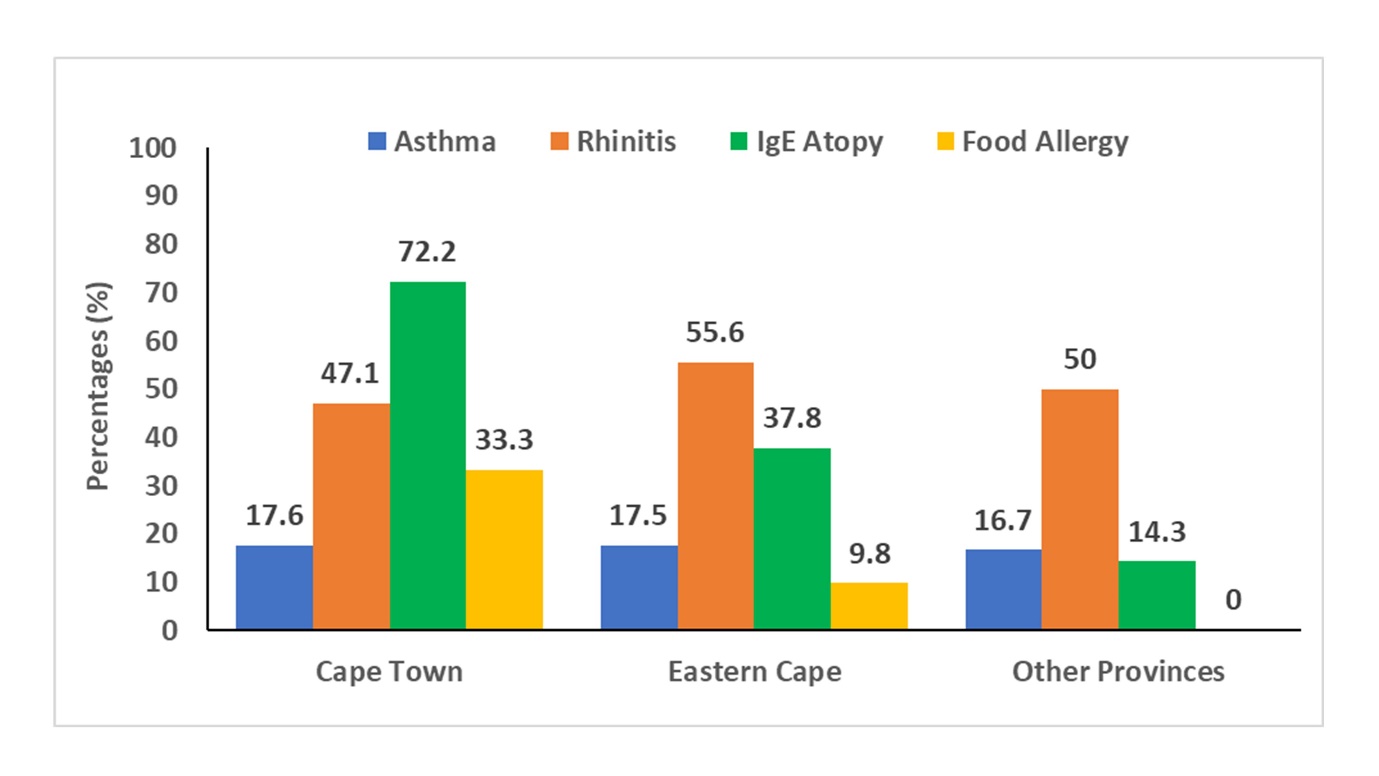
**Supplementary Figure S2. Prevalence of allergic disorders categorized according to participants place of birth.**

**Figure S2 footnote: Cape Town** **(n = 17 for asthma and rhinitis; n = 18 for IgE atopy and food allergy). Eastern Cape (n = 189 for asthma and rhinitis; n = 193 for IgE atopy and food allergy). Other Provinces (n = 6 for asthma and rhinitis; n = 7 for IgE atopy and food allergy). IgE: Immunoglobulin E.**
